# Xanthine urolithiasis: Inhibitors of xanthine crystallization

**DOI:** 10.1371/journal.pone.0198881

**Published:** 2018-08-29

**Authors:** Felix Grases, Antonia Costa-Bauza, Joan Roig, Adrian Rodriguez

**Affiliations:** Laboratory of Renal Lithiasis Research, Faculty of Sciences, University Institute of Health Sciences Research (IUNICS-IdISBa), University of Balearic Islands, Palma de Mallorca, Spain; Universite Paris Diderot, FRANCE

## Abstract

**Objective:**

To identify *in vitro* inhibitors of xanthine crystallization that have potential for inhibiting the formation of xanthine crystals in urine and preventing the development of the renal calculi in patients with xanthinuria.

**Methods:**

The formation of xanthine crystals in synthetic urine and the effects of 10 potential crystallization inhibitors were assessed using a kinetic turbidimetric system with a photometer. The maximum concentration tested for each compound was: 20 mg/L for 3-methylxanthine (3-MX); 40 mg/L for 7-methylxanthine (7-MX), 1-methylxanthine (1-MX), theobromine (TB), theophylline, paraxanthine, and caffeine; 45 mg/L for 1-methyluric acid; 80 mg/L for 1,3-dimethyluric acid; and 200 mg/L for hypoxanthine. Scanning electron microscopy was used to examine the morphology of the crystals formed when inhibitory effects were observed.

**Results:**

Only 7-MX, 3-MX, and 1-MX significantly inhibited xanthine crystallization at the tested concentrations. Mixtures of inhibitors had an additive effect rather than a synergistic effect on crystallization.

**Conclusion:**

Two of the inhibitors identified here—7-MX and 3-MX—are major metabolites of TB. In particular, after TB consumption, 20% is excreted in the urine as TB, 21.5% as 3-MX, and 36% as 7-MX. Thus, consumption of theobromine could protect patients with xanthinuria from the development of renal xanthine calculi. Clinical trials are necessary to demonstrate these effects *in vivo*.

## Introduction

Xanthine urolithiasis is an infrequent type of renal stone formation caused by xanthinuria, a rare hereditary disorder. An affected patient has a deficiency of xanthine oxidase, resulting in hypouricemia and hypouricosuria [1, 2]. Symptom onset can occur at any age. Approximately 50% of patients with classical hereditary xanthinuria present with urinary tract infections, hematuria, renal colic, acute renal failure, and urolithiasis. A small number of patients also develop renal failure, arthropathy, myopathy, or duodenal ulcer [1, 2].

Hereditary xanthinuria is caused by a deficiency of xanthine dehydrogenase/oxidase (XDH/OX), leading to a reduced degradation of hypoxanthine and xanthine to uric acid, and the accumulation of these two uric acid precursors. Classically, Type I xanthinuria is caused by a mutation in *XDH/XO* gene mapped to chromosome 2p23.1, whereas Type II xanthinuria is caused by deficits of XDH/OX and aldehyde oxidase (AO) caused by mutations in molybdenum *cofactor* sulfurase gene (*MOCOS*) localized on chromosome 18q12.2 [3–6]. These different mutations lead to clinically undistinguishable types.

A third clinically distinct entity, molybdenum cofactor deficiency type A (OMIM 252150), is characterized by triple deficiency of XDH, AOX and sulfite oxidase. This rare lethal autosomal recessive disorder caused by mutations in *MOCS1* gene (6p21.1) is characterized by early onset in infancy.

Traditionally, the type of hereditary xanthinuria has been stablished by allopurinol loading test or liver biopsy, because xanthine dehydrogenase/ xanthine oxidase (XDH/XO) activity in humans is expressed only in the small intestine and liver. The modern approach to diagnose and determine the type of xanthinuria is three-step algorithm [7]. First step, xanthinuria is diagnosed by extremely low serum/urinary uric acid which is replaced by xanthine. Second, xanthinuria is typed using urinary metabolomics: N1-methyl-2-pyridone-5-carboxamide (2PY) and N1-methyl-4-pyridone-5-carboxamide (4PY) are the final products excreted in urine in the nicotinamide catabolism and these products are results of the oxidation of N1-methylnicotinamide by aldehyde oxidase (AO)), Finally, the results are confirmed by molecular genetics.

The only recommended treatment for patients with xanthinuria is a low purine diet and high intake of fluids. Because the solubility of xanthine is relatively independent of urinary pH, urine alkalinization has no effect (in contrast to patients with uric acid lithiasis) [8, 9].

There is a need to identify new agents that can prevent the development of xanthine crystals in the urine of patients with xanthinuria.

## Materials and methods

### Reagents and solutions

Xanthine (X), 1-methylxanthine (1-MX), 3-methylxanthine (3-MX), 7-methylxanthine (7-MX), hypoxanthine (HX), theophylline (TP), paraxanthine (PX), theobromine (TB), caffeine (CF), 1-methyluric acid (1-MU), and 1,3-dimethyluric acid (1,3-DMUA) were purchased from Sigma-Aldrich (St Louis, MO, USA). Synthetic urine compounds were obtained from Panreac (Montcada i Reixac, Barcelona, Spain). Chemicals of analytical/reagent-grade purity were dissolved in ultra-pure deionized water from a Milli-Q system, and passed through 0.45 μm pore filters before use. A xanthine stock solution was prepared daily by dissolving 0.5 g of xanthine in 0.1 L of 1 M NaOH. To avoid precipitation of other compounds, such as calcium oxalate or phosphates, crystallization reactions were performed in a simplified synthetic urine, prepared by dissolving 5.60 g Na_2_HPO_4_·12H_2_O, 2.41 g NaH_2_PO_4_·2H_2_O, and 13.05 g NaCl in 1 L H_2_O.

### Turbidimetric assay

Xanthine crystal formation in synthetic urine and the effects of potential crystallization inhibitors were assessed using a kinetic turbidimetric system. This consisted of a photometer (Metrohm 662), a fiber-optic light-guide measuring cell with an attached reflector (light path: 2 × 10 mm), and a monochromatic light source (550 nm). Crystallization was assessed at constant temperature (37°C) with magnetic stirring (300 rpm).

Synthetic urine (180 mL) was added to a crystallization flask, followed by addition of a xanthine solution (20 mL) to a final xanthine concentration of 500 mg/L. When testing an inhibitor, the desired amount was dissolved in this solution. When the resulting solution reached a temperature of 37°C, then 3.6 mL of 6 M HCl was added to achieve a pH of 6.0 (normal urinary pH), and the timer was switched on. The pH of the final solution was measured at the beginning of each experiment, and the absorbance of the solution (550 nm) was recorded during the entire kinetic assay.

The maximum concentration tested for each of the 10 compounds were: 20 mg/L for 3-MX; 40 mg/L for 7-MX, 1-MX, TB, TP, PX, and CF; 45 mg/L for 1-MU; 80 mg/L for 1,3-DMU; and 200 mg/L for HX.

### Analysis of crystal development

A 100 mL aliquot of synthetic urine containing 400 mg/L xanthine at (pH 6.0) was added to 7 crystallization dishes: 1 without inhibitors, 2 with 20 and 40 mg/L of 3-MX; 2 with 20 and 40 mg/L of 7-MX; 1 with a mixture of 20 mg/L of 3-MX and 20 mg/L of 7-MX; and 1 with a mixture of 20 mg/L of 3-MX and 40 mg/L of 7-MX. The dishes were covered with parafilm, and incubated without shaking at 37°C for 24 h. The crystals were carefully collected, dried, and examined by scanning electron microscopy (SEM).

## Results

**Fig 1:**
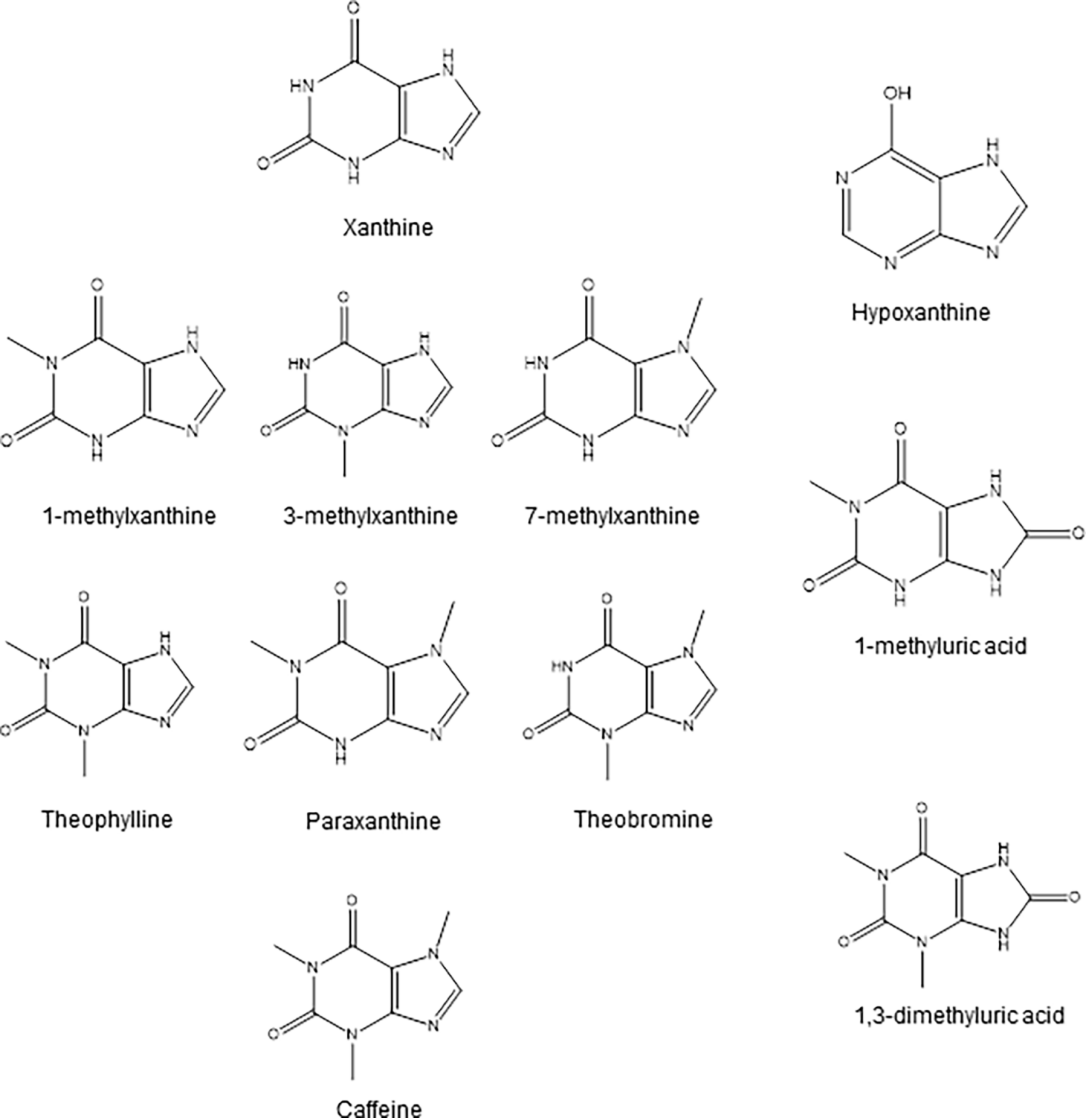
Chemical structure of xanthine and the 10 compounds studied as potential xanthine crystallization inhibitors.

**Fig 2:**
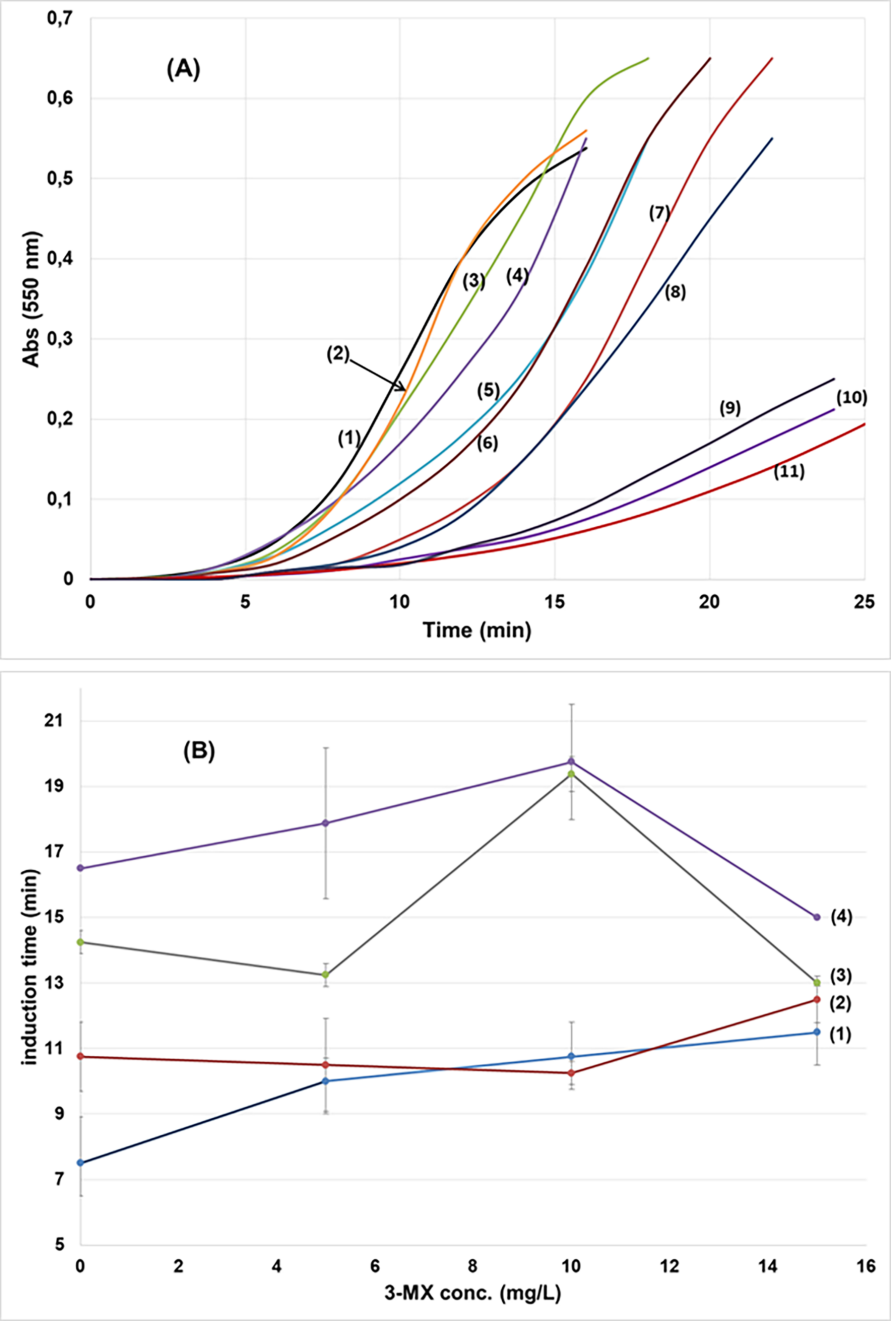
Effects of studied compounds on xanthine crystallization. (A) Crystallization curves for 500 mg/L xanthine in synthetic urine (1) in the absence of inhibitors, and in the presence of 20 mg/L of (2) paraxanthine, (3) caffeine, (4) theobromine, (5) theophylline, (6) 1,3-dimethyluric acid, (7) hypoxanthine, (8) 1-methyluric acid, (9) 1-methylxanthine, (10) 3-methylxanthine and (11) 7-methylxanthine (T = 37°C; pH = 6.0). (B) Effects of 3-methylxanthine + 7-methylxanthine mixtures on the crystallization of xanthine. Induction time ± SD in presence of different concentrations of 3-methylxanthine and (1) absence of 7-methylxanthine, presence of (2) 10 mg/L, (3) 20 mg/L and (4) 30 mg/L of 7-methylxanthine.

We studied the effect of 10 compounds on xanthine crystallization in synthetic urine (Fig 1). Three of these compounds—1-MX, 3-MX, and 7-MX—significantly inhibited xanthine crystallization, and the others had slight or no effects at the highest tested concentrations (Fig 2A). We also examined the possible synergistic effects of 3-MX and 7-MX (Fig 2B). These products were selected because they are major metabolites of TB, while 1-MX is a metabolite only of CF [10]. The results indicated additive, not synergistic, effects.

**Fig 3:**
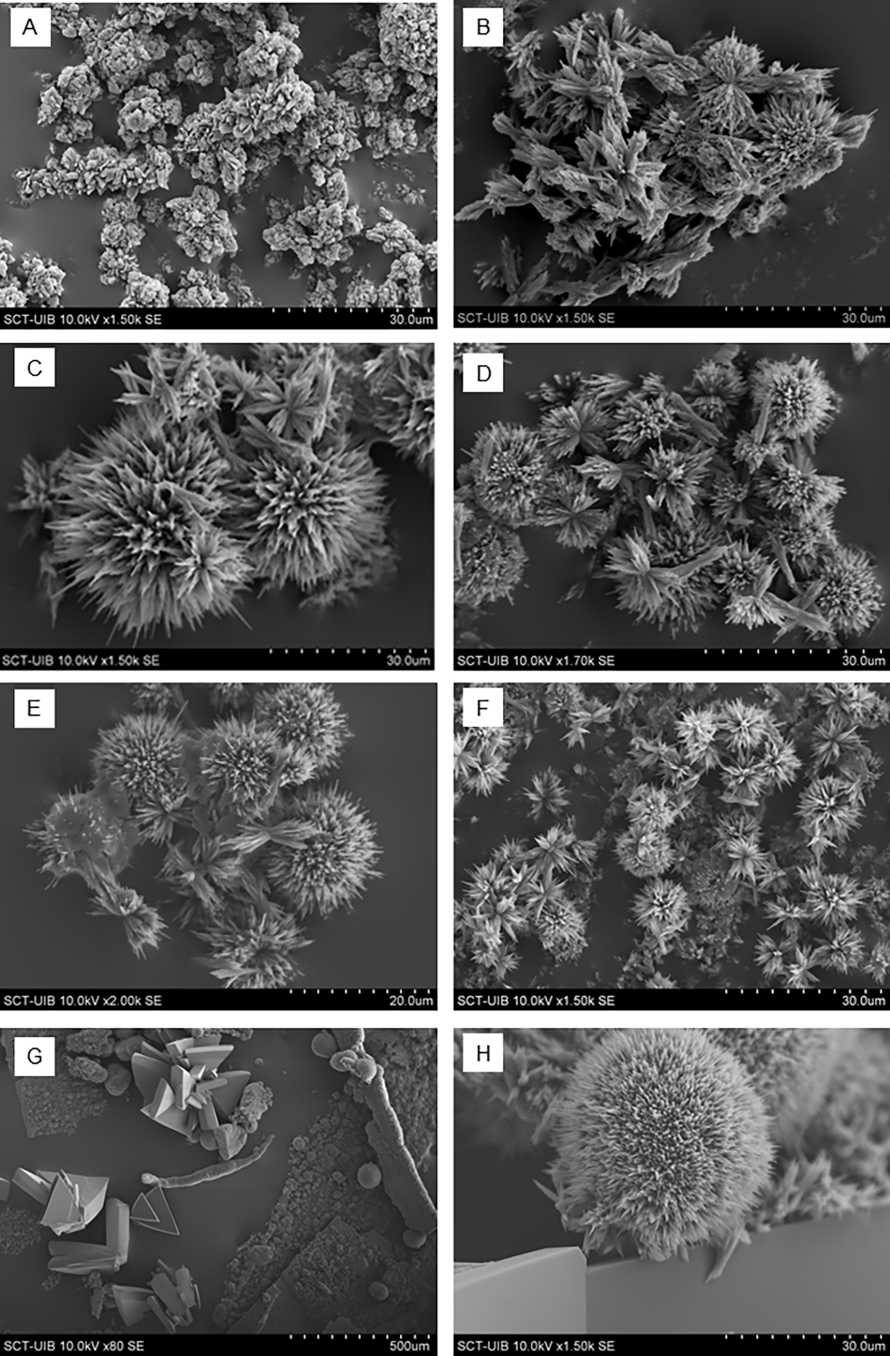
Variation of the morphology of xanthine crystals due to the presence of some methylxanthines. Scanning electron microscopy images of xanthine crystals obtained in synthetic urine containing 400 mg/L xanthine, and incubated 24 h at 37 ^o^C, in the presence of different inhibitors as follows: (A) without inhibitors; (B) with 20 mg/L 3-MX; (C) with 40 mg/L 3-MX; (D) with 20 mg/L 7-MX; (E) with 40 mg/L 7-MX; (F) with 20 mg/L 3-MX and 20 mg/L 7-MX and (G) and (H) with 20 mg/L 3-MX and 40 mg/L 7-MX.

We used SEM to examine the formation of crystals with and without addition of 3-MX, 7-MX, and mixtures of them (Fig 3). The experiments at long time (24 h), show that the crystals obtained in the presence of 3-MX and 7-MX without changes in xanthine supersaturation, are significantly larger than in their absence. This fact demonstrates a clear inhibition of nucleation, since the smaller the number of nuclei formed, the greater the size of the crystals for a given supersaturation and for long time experiments (i.e., when equilibrium is reached).

Thus, our results clearly demonstrate the efficacy of three compounds (1-MX, 3-MX and 7-MX) as inhibitors of xanthine crystallization in *in vitro* experiments.

## Discussion

Our results indicate that 1-MX, 7-MX, and 3-MX significantly inhibited xanthine crystallization *in vitro*. Moreover, the delay in time for induction of crystallization was dependent on the concentration of each inhibitor. It is important to note that HX, TP, PX, TB, CF, 1-MU, and 1,3-DMU had no significant effects on xanthine crystallization. This selective inhibition of xanthine crystallization among these structurally similar compounds is notable. Thus, among the xanthines studied, only methyl xanthines are incorporated into the crystalline network of xanthine. The incorporation of any of these 3 inhibitors into the xanthine crystal lattice modifies the structure of some layers, thereby increasing the Gibbs free energy, and slowing the growth of the crystal. Because xanthine solubility is relatively insensitive to urinary pH, we performed all inhibition studies at a pH of 6.0. Interestingly, TB is also very effective inhibitor of uric acid crystallization [11], although CF, PX, and TP have no effect on uric acid crystallization. This demonstrates the importance of the position of the methyl group on the efficacy of an inhibitor.

It is important to consider that two of the inhibitors of *in vitro* xanthine crystallization identified here are major metabolites of theobromine. In particular, after TB consumption, 20% is excreted as TB, 21.5% as 3-MX, and 36% as 7-MX [10]. In contrast, 1-MX is not a metabolite of TB; instead, after consumption of caffeine, 19% is excreted as 1-MX [10]. TB is present in high amounts in chocolate and cocoa [12], but has been less studied than other methylxanthines because it has less of an effect on the central nervous system than other xanthines. Extrapolation of previous *in vivo* data [10,12] leads to the estimate that daily intake of only 200 mg of TB would lead to excretion of 43 mg of 3-MX and 72 mg of 7-MX. These amounts, in accordance with the results presented here, could significantly inhibit the formation of xanthine crystals in urine, and therefore prevent the development of renal xanthine calculi in patients with xanthinuria.

Our results indicate that two metabolites of TB—7-methylxanthine and 3-methylxanthine—can inhibit xanthine crystallization, and therefore have high clinical potential for prevention of nephrolithiasis in patients with xanthinuria. Clinical trials of patients with xanthinuria are necessary to document the *in vivo* effects of TB consumption.
